# Diagnostic Challenges in Sepsis

**DOI:** 10.1007/s11908-021-00765-y

**Published:** 2021-10-25

**Authors:** Chris F. Duncan, Taryn Youngstein, Marianne D. Kirrane, Dagan O. Lonsdale

**Affiliations:** 1 Department of Critical Care, St George’s University Hospitals NHS Foundation Trust, London, UK; 2 Department of Rheumatology, Hammersmith Hospital, Imperial College Healthcare NHS Trust, London, UK; 3 National Heart and Lung Institute, Imperial College London, London, UK; 4 Department of Intensive Care Services, Royal Brisbane and Women’s Hospital, Brisbane, Australia; 5 Faculty of Medicine, University of Queensland, Brisbane, Australia; 6 Department of Clinical Pharmacology, St George’s, University of London, London, UK

**Keywords:** Infection, Sepsis, Diagnosis, Critical care

## Abstract

**Purpose of Review:**

Sepsis is a leading cause of death worldwide. Groundbreaking international collaborative efforts have culminated in the widely accepted surviving sepsis guidelines, with iterative improvements in management strategies and definitions providing important advances in care for patients. Key to the diagnosis of sepsis is identification of infection, and whilst the diagnostic criteria for sepsis is now clear, the diagnosis of infection remains a challenge and there is often discordance between clinician assessments for infection.

**Recent Findings:**

We review the utility of common biochemical, microbiological and radiological tools employed by clinicians to diagnose infection and explore the difficulty of making a diagnosis of infection in severe inflammatory states through illustrative case reports. Finally, we discuss some of the novel and emerging approaches in diagnosis of infection and sepsis.

**Summary:**

While prompt diagnosis and treatment of sepsis is essential to improve outcomes in sepsis, there remains no single tool to reliably identify or exclude infection. This contributes to unnecessary antimicrobial use that is harmful to individuals and populations. There is therefore a pressing need for novel solutions. Machine learning approaches using multiple diagnostic and clinical inputs may offer a potential solution but as yet these approaches remain experimental.

## Introduction

The third consensus definition of sepsis is ‘life-threatening organ dysfunction caused by a dysregulated host response to infection’ and septic shock a subset of patients with a vasopressor requirement and lactate > 2 mmol/L [1]. At its most severe, sepsis continues to have a high mortality. Agreed criteria to define sepsis are intended to identify patients early, allowing the prompt initiation of treatment, including sepsis care bundles, which may improve outcomes [2]. It also provides a common language for international research and outcome reporting [1]. The need for a *third* consensus definition points to the challenges underlying the diagnosis of sepsis and the evolution of our understanding of the pathophysiology of this disease and its mimics.

The first consensus definition, made in the 1990’s, utilised one haematological and three clinical variables (temperature, heart rate, respiratory rate and white blood cell count) to identify sepsis in those with infection [3]. These variables, which defined the systemic inflammatory response syndrome (SIRS), provided a useful starting point for standardising the diagnosis of sepsis but had some limitations. Foremost among these is that SIRS is found frequently in patients admitted to intensive care units (ICU) regardless of the reason for admission [4–7], yet still fails to identify some patients with severe infection causing significant organ dysfunction [8]. The most recent criteria characterise patients with sepsis as those with a change in SOFA (sequential organ failure assessment) score of 2 points or more within a 24-h time period in the presence of suspected infection. The argument in favour of this change is cited as improved predictive validity over SIRS for ICU mortality or prolonged ICU stay. Further advocated is the identification of  those patients with infection who are at risk of deterioration using clinical criteria (qSOFA) — two or more of altered mental state, tachypnoea (defined as a respiratory rate > 22 breaths per minute) or hypotension (defined as systolic blood pressure < 100 mmHg) [1]. These criteria make the definition of sepsis clear and its diagnosis in the patient with an infection straightforward, since SOFA scores are readily calculable from routine examination and laboratory tests.

What is not easy is the diagnosis of infection. Indeed, the SEPSIS-3 task force specifically state that examining the definitions of infection was not ‘within the task force brief’. Datasets used to model the performance of the qSOFA and the new definition of sepsis contained patients with ‘suspected infection’. There is no commentary on how the suspicion of infection arises or what objective thresholds exist to make a diagnosis of infection, suspected or otherwise. Similarly, the surviving sepsis guidelines seem to take the diagnosis of infection or suspected infection as read. ‘Diagnosis’ comes in third in the order of consideration after resuscitation and screening for sepsis. Commentary on diagnostic and prognostic biomarkers of infection first appear some 18 pages into these guidelines [2].

In this article, we aim to briefly summarise the utility of tools used to aid clinicians in the diagnosis of infection, and therefore in the diagnosis of sepsis.

## Screening for Sepsis

The  2016 iteration of the surviving sepsis campaign guidelines recommends hospitals implement a sepsis screening system for acutely ill, high-risk patients [2]. The utility of sepsis screening tools, and their applicability, continues to be a subject of debate. Identifying the impact of a screening tool alone is challenging, as sepsis care bundles are often implemented alongside screening tools, perhaps explaining why this remains a ‘best practice statement’ in the Surviving Sepsis Campaign Guidelines. For example, in Levy et al.’s study on the impact of the surviving sepsis campaign in 2010, participants were enrolled only if they already had suspected infection and met SIRS criteria for sepsis [9], meaning the impact of the screening tool itself could not be reviewed. Other data cited by the Surviving Sepsis Campaign arise from quality improvement projects. Jones et al. reported a 12% absolute reduction in sepsis associated mortality after the introduction of a 17-point sepsis screening tool [10], but there was promotion of specific interventions following screening in this study too. Damiani et al. undertook a systematic review of such improvement programmes and found consistent reporting of improvement in sepsis outcomes [11].

The SEPSIS-3 task force noted that a patient found to meet qSOFA criteria should ‘prompt consideration of possible infection in patients not previously recognised as infected’ [12]. One potential drawback of qSOFA as a potential screening tool is that it was identified and tested using a dataset of patients already suspected of having infection [13]. The UK national screening committee notes that the purpose of screening is to offer a test to a population who ‘do not necessarily perceive that they are at risk of, or are already affected by, a disease or its complications’ [14]. Since qSOFA was initially validated in a group of patients in hospital with suspected infection and continues to be tested largely in this population, it arguably falls short on this definition. Indeed, members of the task force have subsequently stated that qSOFA is not a screening tool but should be used to identify those at risk of worse outcome from infection. Given the tools used by clinicians to diagnose infection, it may be preferential to use the formal definition of sepsis to predict poor outcome, since many of these tools will also calculate a formal SOFA score. Perhaps the exception is access to a readily available PaO_2_ for patients outside critical care, although conversion models exist to substitute oxygen saturations (SpO_2_) [15].

## Can Clinicians Agree on Whether an Infection Is Present?

Table 1 provides brief details of presenting symptoms of three imagined case scenarios. They are intended to describe a typical presentation of acute pancreatitis (case 1) and sepsis secondary to a complicated urinary tract infection (case 2). The details provided meet the qSOFA screening criteria that suggest we consider sepsis *if* we suspect infection. We intend to provoke suspicion of infection in both cases, noting that it appears more likely in case 2. Scenario 3 would satisfy the definition of sepsis; however, it is a case of systemic juvenile arthritis with evolving haemophagocytosis with no infectious trigger. We recognise that there will not be universal suspicion of infection and sepsis amongst physicians reading such case examples. In a survey of intensivists in 104 US hospitals, Stevens et al. found significant discordance in diagnosing pneumonia, with some physicians identifying pneumonia in 100% and others 0% of case vignettes presented to them [16]. Lopansri et al. found only moderate inter-observer agreement on the diagnosis of sepsis or SIRS in a multi-centre cohort of patients with culture positive infection, disagreement was most pronounced in cases of respiratory infections [17], and Rhee et al. found similar disagreement in their survey of US clinicians [18]. Similar results have been found in paediatric studies. Peltola et al. found physician’s sensitivity of 38% for diagnosing influenza in a prospective study of 2288 children with respiratory infections [19] and in a prospective cohort study of nearly 16,000 children with fever presenting to a hospital in Australia, Craig et al. found a low sensitivity of physicians’ diagnosis of bacterial infection (10–50%) [20].

**Table 1:** Details of presenting symptoms of three imagined case scenarios

Scenario 1 A 54-year-old man with epigastric pain	Scenario 2 A 78-year-old woman with dysuria and frequency	Scenario 3 A 20-year-old woman with fever and rash
• 10-day history of epigastric pain and nausea • Evolving breathlessness in the 48-h preceding presentation • History of alcohol induced pancreatitis and ongoing alcohol excess • Temperature 38.5 °C, respiratory rate 28 bpm, HR 115 bpm, SpO_2_ 88% breathing air, blood pressure 94/65 mmHg • Inspiratory crackles and epigastric tenderness were found on examination	• 5-day history of suprapubic pain and dysuria • Evolving fatigue and dizzyness in the 48-h preceding presentation • Temperature 39.2 oC, respiratory rate 24 bpm, HR 133 bpm, SpO2 94% breathing air, blood pressure 78/55 mmHg • Bedside urinalysis positive for nitrite, leukocytes and blood	• 14 day history of daily fever to 39 and fatigue • Effervescent pink rash on chest wall – non-blanching • Increasing shortness of breath over 48 h • Reduced urine output • Temperature 39.5, Sp)2 93% on air, RR 30, HR 110, BP 84/65 • History of joint pain as a child with steroid injection
***Initial investigations*** • White cell count 18.3 × 10^9^/L (4–11) • Neutrophil count 12.6 × 10^9^/L (1.5–8.0) • CRP 128 mg/L (0–5)	***Initial investigations*** • White cell count 23.9 × 109/L (4–11) • Neutrophil count 16.5 × 109 /L (1.5–8.0) • CRP 186 mg/L (0–5)	***Initial investigations*** • White cell count 31 × 109/L (4–11) • Neutrophils 29 × 109/L (1.5–8.0) • CRP 338 mg/L

The reader may, or may not, consider infection possible in case 1, but suspicion of infection in cases of acute pancreatitis is high amongst clinicians and antibiotics are often prescribed [21]. The use of routine antibiotics in severe acute pancreatitis is not recommended as there is no evidence of efficacy, either in terms of mortality or reduction in the incidence of infected necrosis [22]. However, as there is some evidence of efficacy in necrotising pancreatitis, international guidelines recommend the use of antibiotics where infection is suspected [23]. In what is a somewhat recurring theme in this subject area, no recommendations are given as to *when* or *how* to suspect infection. Given the most common cause of death in acute pancreatitis *is* infection [24], clinicians are left somewhat in a quandary with regards to antibiotics in this condition. The Surviving Sepsis Campaign guidelines recommend the initiation of antibiotics within one hour of a diagnosis being made or suspected. It seems likely that this time limit will expire prior to any complex cross-sectional imaging can be organised and reported, meaning antibiotics are likely to be given before a diagnosis of necrotising pancreatitis can be confirmed or ruled out.

## Diagnostic Value of Common Laboratory Biomarkers

Blood culture is the gold standard for diagnosis of blood stream infection but is limited by poor sensitivity and the required processing time. Challenges in clinical diagnosis and requirement for prompt diagnosis and treatment have led to reliance on biomarkers such as white blood cell count, C-reactive protein (CRP), and procalcitonin (PCT). Indeed, the first consensus sepsis definition incorporated an increased (> 12 × 10^9^ /L) or decreased (< 4 × 10^9^ /L) white blood cell count into the SIRS criteria [3]. However, the utility of serum biomarkers to predict or exclude blood stream infection is debated, with many of the commonly used tests lacking sensitivity and specificity. In a recent retrospective cohort study, Marik and Stephenson reported white blood cell count to have a very poor predictive value for bacteraemia in patients presenting with suspected sepsis with an area under the receiver operating characteristic (AUROC) as low as 0.52 [25]. Siegel et al. found that 52% of patients presenting to the Emergency Department with proven blood culture positive bacteraemia had a normal white blood cell count as defined by the SIRS criteria [26]. In a meta-analysis of the value of diagnostic tests in febrile children, Van den Bruel et al. found white cell count to be of little diagnostic benefit in ruling out serious infection, with a negative likelihood ratio as low as 0.61 [27]. In a retrospective analysis of 1169 appendicectomies in a district general hospital, Panagiotopoulou et al. found neither raised CRP or white cell count as useful diagnostic markers for acute appendicitis. Whilst they found a raised white cell count to be relatively sensitive (84%), it was not specific (58%) and did not have a favourable negative predictive value (68%). The results were similar for CRP [28]. Warschkow et al. found similar results in their review of the routine blood tests of patients who had undergone open resection of colorectal cancer [29]. They found day 5 raised white cell count to have a sensitivity of 70% and specificity of 58% for inflammatory complications.

The neutrophil to lymphocyte count ratio has been consistently reported to be a more accurate marker of physiological stress than absolute white blood cell or neutrophil counts [30, 31]. A rise of neutrophil count and fall in lymphocyte count is commonly encountered in systemic illness and is hypothesised to be due to the endogenous actions of cortisol and catecholamines. Furthermore, sepsis causes lymphocyte migration to inflammatory tissues and increased lymphocyte apoptosis resulting in a greater rise in neutrophil to lymphocyte count ratio when compared with other causes of physiological stress. A normal neutrophil to lymphocyte ratio is reported to be 2.15 and a level of 10 considered to be a threshold for the diagnosis of bacteraemia [32, 33]. In a prospective study of 1572 patients presenting to the Emergency Department, Ljungstrӧm et al. found neutrophil to lymphocyte count ratio to be superior to PCT and CRP for the diagnosis of bacterial sepsis (AUROC 0.68 vs 0.64 vs 0.57, *p* < 0.05) and the diagnosis of bacterial infection, although the difference in AUROC did not reach statistical significant in the latter [34]. Neutrophil to lymphocyte count ratio is seen to be elevated in any form of severe physiological stress and is not specific for the diagnosis of bacteraemia. This is recently evidenced by its application for predicting disease severity in patients with coronavirus disease 2019 (COVID-19) [35]. In addition, neutrophil to lymphocyte count ratio is significantly less accurate in the diagnosis of sepsis in the critical care population in whom there is invariably an elevated neutrophil to lymphocyte count ratio even in non-infected patients. Westerdijk et al. reported neutrophil to lymphocyte count ratio to have an AUROC of 0.66 for predicting sepsis in critical care versus remarkably high AUROCs of 0.89 and 0.88 for CRP and PCT, respectively [36].

C-reactive protein (CRP) is an acute phase protein synthesised in the liver following inflammatory stimuli, with concentrations rising within 12–24 h [37, 38]. Although a commonly used marker in critical illness, CRP lacks specificity for bacterial infection and is seen to rise in most other causes of inflammation. Serum CRP level may take up to 72 h to peak, contributing to diagnostic and surveillance challenges [39]. In a meta-analysis assessing the diagnostic accuracy of CRP in sepsis, Tan et al. found CRP to have a AUROC of 0.75 with a pooled sensitivity of 0.80 but a specificity of only 0.61 [40]. The level of CRP was reported to have little correlation with the severity of illness in sepsis. Conversely, CRP is the most utilised biomarker for predicting disease severity in pancreatitis due to its low cost and widespread availability [41]. A CRP level of > 150 mg/L is regarded as a threshold marker of pancreatitis severity with Khanna et al. reporting this cutoff level to have a 100% sensitivity and 81.4% specificity for necrotising pancreatitis [42]. CRP does not, however, consistently distinguish between sterile and infected pancreatic necrosis and is not recommended to be used as a marker to commence antimicrobial therapy. The CRP trend between two different time points is more efficacious with Póvoa et al. reporting a daily CRP variation of > 41 mg/L in critical care patients predicting bacterial infection with a sensitivity and specificity of 0.92 and 0.71, respectively [43]. Erythrocyte sedimentation rate (ESR) is an alternative non-specific test of systemic inflammation that remains in use in some centres, particularly for the surveillance of rheumatological disorders. It demonstrates a slower rise than CRP and has been suggested to be inferior to PCT, CRP and WBC count in the diagnosis and prognosis of sepsis using the SEPSIS-3 criteria [44].

Procalcitonin (PCT) is a precursor to calcitonin. Its production is upregulated in response to sepsis and has been suggested to reliably distinguish between bacterial infection and other inflammatory states [45]. PCT rises 2–3 h following infection and reaches a peak at 24 h. Although a faster rise than CRP, the delay in reaching peak concentration necessitates caution when using PCT as a sole marker of infection at initial presentation. Highest PCT levels are detected in gram-negative bacteraemia with only a minimal elevation in fungal infection [46]. Given the increasing prevalence of fungaemia, PCT is a potentially useful biomarker to predict those who will not benefit from empirical antifungal therapy [46–48]. In a systematic review and meta-analysis, Wacker et al. reported PCT to be a good biomarker to differentiate between sepsis and other non-inflammatory syndromes with an AUROC of 0.85 [49]. Pooled results from the several meta-analyses have reported overall sensitivity and specificity ranges of 0.72 to 0.93 and 0.64 to 0.84, respectively, with positive and negative likelihood ratios of 3.0 to 5.9 and 0.11 to 0.44 [50]. These data suggest a moderate discriminatory ability between bacterial infection and non-infectious inflammatory states but a negative test cannot exclude infection. PCT may be useful in predicting infective versus non-infective pancreatic necrosis with Rau et al. reporting a threshold of > 1.8 ng/mL having a similar sensitivity (0.95) and specificity (0.88) to US guided fine needle aspiration (FNA) [51]. Although subsequent research predicts a slightly lower sensitivity, these data have prompted international guidelines to suggest using PCT to aid in deciding whether to commence antimicrobial therapy [52, 53]. PCT may be less effective in distinguishing between bacterial and viral infection with Kamat et al. reporting poor sensitivity (0.55) and only moderate specificity (0.76) in a systematic review and meta-analysis of 12 papers focused on serum PCT levels in patients with community-acquired pneumonia [54]. Similarly, PCT exhibits a lower diagnostic AUROC and sensitivity for predicting bacterial infection in patients with renal impairment (defined by an estimated glomerular filtration rate < 30 mL/min/1.72 m^2^) [55], immunocompromised patients [56] and in patients with autoimmune conditions [57].

Numerous studies have assessed the safety and efficacy of discontinuing antimicrobial therapy when PCT concentration reaches < 1 ng/mL or falls by 65–90% from peak values. They have demonstrated a reduction in total duration of antimicrobials (around 2 days) without a negative impact on length of stay and mortality rates [58–60]. Many of the antimicrobial courses in the PCT-guided groups remained > 7 days emphasising that more evidence is required to correlate this data with emerging evidence that shorter antibiotic courses may be appropriate for certain disease states [61].

The initial investigations in our theoretical cases emphasise the non-specific nature of commonly used biomarkers. The history consistent with urinary tract infection and raised inflammatory markers is highly predictive of infection in case 2 with indicators of severe disease (e.g. hypotension) that may indicate sepsis. Similarly, the findings and investigations in the other scenarios also support the diagnosis of a significant inflammatory insult that would prompt many clinicians to consider infection and prescribe empirical broad-spectrum antimicrobials for fear of missing the *1-h window*. In scenario 1, although the CRP has not exceeded the severity threshold of > 150 mg/L, the white blood cell count level would contribute to severity prediction using both the Glasgow-Imrie and Ranson’s criteria [62, 63]. Readers may or may not consider PCT to be useful in this case, but it is not commonly performed within the first hour of presentation. Similarly, in scenario 3, findings may suggest infection (and sepsis), although all cultures were consequently negative and a significantly elevated ferritin (56,000 µg/L [10-120]) favours a diagnosis of Still’s disease [64].

## Microbiological Diagnosis of Infection

In the absence of a suitable alternative test, detection of pathogens from blood culture samples remains the gold standard for diagnosing blood stream infection. Unfortunately, routine blood cultures may take over 72 h to yield a detectable organism with further time required to identify and test for antibiotic susceptibility. This timeframe is unacceptable in sepsis where delays in treatment worsen morbidity and mortality and contributes to the need for empirical, non-targeted antibiotic therapy [65]. A recent meta-analysis of seven studies including 22,655 patients with sepsis or septic shock identified that only 40.1% of patients had positive blood cultures [66]. This is more pronounced in the neonatal population where, even in symptomatic neonates, only 10 to 15% of blood cultures yield a positive causative result after excluding contaminants [67]. A multitude of factors may contribute to this poor diagnostic yield. For example, the lack of reliable diagnostic criteria for infection in sepsis means that many patients actually have non-infectious inflammatory states due to metabolic, neurological or inflammatory disorders [68]. Many patients are administered antibiotics prior to the development of or worsening of sepsis and prior to blood culture sampling. Cheng et al. demonstrated an absolute difference in the proportion of positive blood cultures between pre- and post-antimicrobial testing of 12.0% [69]. This reduces the probability of pathogen detection [70]. Finally, many common pathogens, including some bacteria, viruses and fungi, are not detectable using conventional culture techniques and rely on surrogate markers such as urinary antigens and non-specific fungal markers. Given the growing incidence of sepsis caused by atypical organisms this may become increasingly problematic [71].

The culture of other fluids and samples, such as urine, sputum, cerebrospinal fluid, faeces, wound and skin swabs, are subject to the same significant time delays as blood culture making them unsuitable for the initial diagnosis of bacterial infection. Essentially *any sample* may be cultured to identify a causative organism but the results are very variable and often do not yield positive results even in the presence of severe sepsis (a now redundant term) [72, 73]. Simple bedside diagnostic tests tend to lack reliability to exclude infection. Mambatta et al. found the presence of nitrite and leukocyte esterase on urine dipstick analysis to only have a sensitivity of 23.31% and 48.5% respectively [74]. Sputum gram stain has been found to be useful in the aetiologic diagnosis of community acquired pneumonia but is not routinely used and further work is needed to ascertain the impact on clinical outcomes [75].

Novel techniques for pathogen detection may offer an encouraging alternative to conventional methods. Matrix-assisted laser desorption ionization time-of-flight mass spectrometry (MALDI-TOF MS) is a rapid diagnostic technology capable of accurately identifying bacteria, yeast, fungi, Nocardia and mycobacteria species as soon as growth on culture is detected [76]. It has been demonstrated to hasten the identification of pathogens by 17 to 34 h, thereby reducing the time taken for effective and optimal antimicrobial strategies in sepsis [77–83]. These studies also found that MALDI-TOF MS decreased the length of hospital stay by 1.75 to 6 days [78, 82] and improved overall survival by 4 to 9% [78, 83], emphasising the importance of early pathogen recognition. MALDI-TOF MS is currently unable to detect antimicrobial resistance mechanisms and therefore testing antibiotic susceptibility relies upon traditional laboratory techniques [76]. Research to identify spectral peaks that correlate with enzymatic mechanisms of antimicrobial resistance is ongoing and will further increase the value of this technology for tailoring antibiotic therapy [84, 85].

New systems incorporating the polymerase chain reaction (PCR) for microbe amplification, prior to mass spectrometry (MS) detection, have been developed to rapidly accelerate the identification of clinically relevant bacteria and yeast species (turnaround time advertised as 6 to 8 h) with a higher diagnostic yield than blood culture [86]. Furthermore, microorganisms that do not reliably grow in blood cultures may be detectable including *Legionella pneumophila, Mycoplasma pneumoniae*, *Rickettsia typhi*, *Nocardia* spp. and various fungi [87]. Makristathis et al. additionally reported that following prior antimicrobial therapy, positive results were detected in 82.9% of patients with PCR/MS versus only 41.5% with blood cultures [88]. PCR methods enable detection of co-infection with bacteria and viruses which is a frequent phenomenon in community acquired pneumonia [89]. Initial concerns regarding *over-sensitivity* of these tests due to PCR amplification have been addressed by introducing semi-quantitative methods with an aim to identify and eradicate contaminants. Although commercially available and useful for complementing and expediting microbiological diagnosis of sepsis, these techniques have not yet consistently reached sufficient positive predictive value to replace conventional cultures [90]. They are also unable to identify more than a handful of markers of antibiotic resistance which is an essential factor in providing targeted therapy [91]. Although a promising step forward, these techniques have not yet reached the desired target of accurate detection of organisms with antibiotic susceptibility within a 1 to 3-h window to allow targeted initial management.

PCR has been used in detection of viruses since the late 1980s. For RNA viruses, a reverse transcriptase (RT) process is required to convert the single stranded RNA into complementary DNA. RT-PCR is the frequently used method for detecting viruses due to its low cost, simplicity and high sensitivity [92]. It is limited by a high false positive rate due to contamination and the ability of RT-PCR to detect fragments of non-viable viruses from a previous illness. Real-time quantitative polymerase chain reaction (RT-qPCR) has become the gold standard for virus detection and facilitates quantification of sequences by measuring fluorescence emission during the amplification stage. This provides a high sensitivity and low detection limit [93]. In a sample size of 519 people, Sundell et al. reported the detection of respiratory viruses in asymptomatic individuals was as low as 4.3% from nasopharyngeal swabs suggesting that positive results in symptomatic individuals are likely to be significant [94]. Although bacteria are the predominant causative pathogen for sepsis [95], viruses are often underdiagnosed with several studies detecting viral infection in up to one-third of adults with septic shock [96, 97]. Whilst there is no current recommendation for the use of empirical antiviral therapy in sepsis, it may be prudent to screen for viral infection in patients with severe respiratory illness. This strategy has been widely applied during the COVID-19 pandemic which has mandated the widespread use of real-time PCR testing. Due to the rapid spread of the SARS-CoV-2 virus, rapid antigen lateral flow tests have provided an alternative testing strategy to deliver results in minutes rather than the several days required for PCR in most institutions. The low cost, speed and ease of use of antigen testing is an attractive method for guiding patient management and public health decisions but they lack the sensitivity of PCR, and many have not undergone stringent regulatory review. WHO guidance advises confirmation of positive tests using conventional PCR testing [98].

## Future Role of biomarkers, machine learning and gene expression

The absence of an ideal infection or sepsis biomarker has led to the research of hundreds of novel tests [50, 99]. The most extensively investigated include IL6 [100], CD64 [101], presepsin [102], calprotectin [103], sTREM-1 [104] and pentraxin-3 [105]. Unfortunately, many of these tests exhibit the same limitations as conventionally used biomarkers and none have been robustly proven to be superior [99]. Presepsin and CD64 are the most promising biomarkers and are suggested to have a greater sensitivity and shorter time to peak levels than conventional biomarkers although larger studies are required [39, 106]. Studies into novel biomarkers are limited by diverse methodology and small study size with many only having been assessed in populations of < 300 patients. Larger, multi-centre studies are required that account for the heterogeneity of sepsis, before any of these tests change global clinical practice.

Combining several biomarkers has been shown to improve the diagnostic accuracy in sepsis [107, 108]. Han et al. demonstrated the combination of PCT and CRP to have a high ability to discriminate between bacterial sepsis and non-infectious inflammation in the ICU [109]. In a systematic review and meta-analysis including 2661 patients, Ruan et al. reported the same combination to have a higher sensitivity (0.94), AUROC (0.96) and lower negative likelihood ratio (0.89) than CRP or PCT alone in the diagnosis of neonatal sepsis [110]. Similarly, the combination of CD64 and CRP was found to be more sensitive for neonatal sepsis than either measure alone [111]. Combination biomarker panels may also perform better than single biomarkers in predicting prognosis in septic patients [112, 113]. The integration of clinical information into these biomarker panel algorithms may further improve the ability to differentiate between sepsis and non-infectious inflammatory states. In a multi-centre prospective study, Mearelli et al. developed a predictive algorithm incorporating age, SOFA score, recent antimicrobial therapy, hyperthermia and a biomarker panel (white blood cell, CRP, PCT, presepsin, soluble phospholipase A2 group IIA & soluble interleukin-2 receptor α) which demonstrated an high negative predictive value for ruling out sepsis in the Emergency Department [108]. The same authors subsequently demonstrated this algorithm to be superior to qSOFA alone in the prognostication of patients with sepsis [114].

The widespread application of electronic medical records (EMR) in modern clinical practice provides a unique opportunity to analyse large amounts of data within machine learning models. The combination of novel biomarkers with EMR data is suggested to be capable of the early diagnosis and differentiation of sepsis from its mimics [115]. A recent meta-analysis by Islam et al. reported machine learning approaches to perform better than conventional scoring systems for predicting sepsis [116]. Machine learning models are described as being either *left-aligned* or *right-aligned*. Left-aligned models are used to predict the onset of sepsis from a fixed point in time — for example from the arrival of the patient into the Emergency Department [117]. The predominant aim of this approach is to expedite the diagnosis and initiation of sepsis treatment to avoid delay. Right-aligned models use large amounts of data to continually predict the development of sepsis following a distinct period of time — effectively the ability to diagnose sepsis before it is clinically evident [118]. The potential value of machine learning algorithms is clear. Fleuren et al. have recently demonstrated in a systematic review and meta-analysis that machine learning models may accurately predict sepsis ahead of time when analysing *retrospective data* [118]. Unfortunately, significant heterogeneity exists in study methodology, reducing the clinical utility of these models. Additionally, most models have been tested using the MIMIC database which is a freely available clinical database of patients from the Beth Israel Deaconess Medical Center in Boston, USA [119]. This potentially means the models have limited applicability in low- and middle-income countries. Nevertheless, this is an area that is likely to expand dramatically in the coming years. Prospective trials are required to confirm the clinical utility of these models.

The role of host gene response to sepsis is another avenue of research to identify a *sepsis signature*. These techniques aim to identify specific RNA biomarkers that arise in response to sepsis but not other causes of inflammation. Two such assays are commercially available, the SeptiCyte Lab [120] and the Sepsis MetaScore [121], which have recently been prospectively validated in the ICU population. The AUROC results for diagnosis of sepsis versus non-infectious inflammation was 0.8 for the Sepsis MetaScore and 0.68 for the SeptiCyte Lab [122]. Although this method is not routinely deployed, these findings are encouraging for the role of precision, gene-based medicine in the future of sepsis treatment.

## Role of Diagnostic Imaging in Sepsis

Identifying the source of sepsis using diagnostic imaging is essential for providing targeted antimicrobial therapy, disease surveillance and interventional source control. The source of sepsis can guide the clinician to the likely causative pathogen and guide antibiotic class and subtype depending upon regional resistance patterns. In the critically unwell patients, conventional history providing clues as to the origin of infection is often unavailable leading to the reliance on *septic screens* including microbiological cultures and diagnostic imaging. Imaging will also enable diagnosis of sepsis mimics, such as acute pulmonary embolus and acute pancreatitis, which require alternative management and can avoid the unnecessary administration of antimicrobials. The optimal imaging modality is dependent upon the suspected source and balanced against the risks of cumulative radiation exposure and moving critically unwell patients.

The chest radiograph (CXR) is the most commonly performed radiological investigation in sepsis [123] which is unsurprising considering respiratory infections are by far the most common source [95]. In many centres, CXR is regularly performed to confirm the position of medical devices, such as nasogastric tubes and central venous catheters, allowing for the incidental identification and surveillance of pathology. Unfortunately, many features of pulmonary consolidation are non-specific for infection and the quality of portable imaging in unwell patients further worsens its performance [124]. Airspace opacities on CXR in ICU may reflect infection, atelectasis, oedema, haemorrhage, inflammation, drug or transfusion reaction or acute respiratory distress syndrome [125]. Winkler et al. in a meta-analysis including 543 patients found CXR to only have a sensitivity of 49% for identifying causative pathology in the ICU [126]. Additionally, the risk of cumulative radiation exposure continues to be a concern in patients with long term critical illness. Machine learning has been reported to increase the diagnostic capability of CXR although is not widely applied. In a systematic review and meta-analysis, Li et al. reported deep learning algorithms to perform with high accuracy for the detection of pneumonia with an AUROC of 0.99 and the ability to differentiate viral from bacterial pneumonia with an AUROC of 0.95 [127]. Recent studies have applied machine learning to CXR interpretation in the COVID-19 pandemic with encouraging results [128].

Recent technological advances in the quality and portability of ultrasound devices have led to the widespread use of point of care ultrasound to assess the lung parenchyma. Several systematic reviews and meta-analyses have found lung US to be superior to CXR for the diagnosis of pneumonia [126, 129–133]. Serial assessments may be performed without repeated exposure to ionising radiation. However, ultrasound is limited to the identification of pathology extending to the lung peripheries due to the high acoustic impedance of air upon ultrasound waves. Similarly, ultrasound is highly user dependent, requires dedicated training and is time consuming for busy clinicians leading to continued reliance on CXR and computerised tomography (CT) imaging [134].

Ultrasound is recommended as the first line imaging modality in suspected biliary tree pathology [135]. It is reported to have a sensitivity of 81% for the diagnosis of acute cholecystitis and 85–95% for the detection of biliary dilatation [136, 137]. Only if the ultrasound is equivocal should further imaging such as magnetic resonance cholangiopancreatography (MRCP) or cholescintigraphy be performed.

Ultrasound, in the form of echocardiography, is the imaging modality of choice in suspected infective endocarditis. 2D transthoracic echocardiography (TTE) is invariably the initial investigation due to being non-invasive, low cost and portable with a sensitivity of 70% and specificity of 90% for native valve infective endocarditis [138, 139]. Transoesophageal echocardiography (TOE) is superior to TTE with a sensitivity of 96% but at the expense of being an invasive procedure with significant potential complications [138, 140]. The sensitivity of both modalities falls in the setting of prosthetic valve endocarditis (TTE 50% versus TOE 92%) but it remains an important investigation to assess ventricular size, performance and valvular dysfunction [141]. Whilst a normal TOE is highly suggestive of a negative diagnosis of endocarditis, it cannot unequivocally be excluded and current guidance recommends that a repeat TOE should be performed after 7–10 days in cases with a high index of suspicion [142]. Diagnosis may be particularly challenging with prosthetic valves (infective endocarditis versus thrombus versus pannus), intracardiac devices, small vegetations, abscesses and in patients with pre-existing valvular lesions such as mitral valve prolapse or degenerative calcific disease. In challenging cases, modern alternative imaging techniques may be deployed such as multi-slice computed tomography (MSCT), magnetic resonance imaging (MRI), ^18^F-fluorodeoxyglucose (FDG) positron emission tomography (PET)/CT or other functional imaging techniques [139].

CT has emerged as the gold standard investigation for many potential sources of infection. It is highly sensitive and specific for the diagnosis of pneumonia and enables differentiation of infective patterns including focal, lobar, bronchopneumonia, interstitial, ground glass, nodular, tree-in-bud and halo sign opacities. These pattern subtypes may provide clues as to the likely causative organisms and guide further investigation [143]. CT is suggested to reduce the *overdiagnosis* of pneumonia when compared with CXR, therefore lowering the inappropriate administration of antimicrobial agents [144]. Incidental radiological findings, such as pulmonary nodules, may be seen in up to one third of elderly patients undergoing imaging for pneumonia providing an opportunity to diagnose and treat unexpected pathology [145]. In a case series of patients with CXR diagnosed community acquired pneumonia admitted to the ICU, Karhu et al. found CT to yield new findings in 58.5% of patients. Of these patients, 76.5% subsequently underwent further diagnostic or therapeutic interventions based upon the CT findings [146].

CT is the diagnostic modality of choice for peritonitis. In a retrospective analysis of 251 patients with post-operative sepsis, Bader et al. found CT to have a diagnostic sensitivity of 97.2% when compared with conventional radiography (66.2%) and US (44.3%) [147]. It can readily identify intra-abdominal infection in ICU patients who have sustained major trauma; a cohort who invariably have signs of systemic inflammatory response with or without infection [148]. Furthermore, CT can accurately identify intra and retroperitoneal collections and guide drainage to avoid the requirement for invasive surgery. Although the safety and success rate of percutaneous drainage of intra-abdominal collections was conventionally considered to be safer with CT compared with ultrasound [149], many modern techniques will use both modalities to enhance outcomes [150]. CT-guided fine needle aspiration (FNA) is the gold standard for diagnosing infected pancreatic necrosis but is not frequently deployed due to the high rate of false negative findings [52]. In the context of severe acute pancreatitis, retroperitoneal gas is considered to be indicative of infection but has a low negative predictive value [151]. This further adds to the dilemma of diagnosing pancreatic infection and promotes the excessive use of empirical antimicrobials.

The high diagnostic utility of CT must be balanced against the risk of transporting acutely unwell patients and the cumulative risk of cancer due to radiation exposure. Radiation dosage depends upon the type of CT but is estimated to range from 1–15 millisieverts (mSv) [152]. The use of CT imaging in children has been found to triple the risk of developing leukaemia and brain tumours [153, 154]. In adults, much of the large population data suggesting the increased cancer risk due to low-level radiation exposure is derived from atomic bomb survivors [155] and workers in the nuclear industry [156]. For context, these sub-groups received radiation doses ranging from 5 to 20 mSv which is the equivalent of 1 to 2 CT scans. Radiation is thought to contribute to 2% of all deaths in this group. Based upon these findings, Shao et al. conducted a retrospective cohort study assessing 56,050 cases of thyroid cancer, leukaemia and non-Hodgkin lymphoma (NHL) in Taiwan and identified a small but significant increase in risk in patients under 45 exposed to medical radiation [152]. The odds ratios for thyroid cancer, leukaemia and NHL for all populations were 2.55, 1.55 and 1.05, respectively. There was a threefold increase in NHL in individuals aged under 45. Whilst the individual risk is low, the increased prevalence of CT imaging is likely to have an impact at a public health level.

FDG-PET/CT has emerged as a useful second line investigation in patients with sepsis of unknown origin and *Staphylococcus aureus* bacteraemia. Areas of active infection are identified as abnormal areas of glucose metabolism due to increased glycolysis by activated white blood cells [157]. FDG-PET/CT exposes the patient to less radiation than a CT scan of the abdomen and pelvis with contrast and can identify many potential foci of infection within one investigation. Whilst potentially appealing, its use is limited by cost and required reporting time. The cost of PET/CT in the UK is £909 compared with around £140 for a CT scan [158]. The diagnostic advantage and cost-effectiveness will need to be justified prior to PET/CT being offered on a routine basis. Recent statistics suggest that the median timeframe from imaging request to being performed is 7 days with a further 2 days required for reporting [159]. This delay is likely to be considered unacceptable for critically unwell patients and promotes continued reliance on conventional imaging techniques.

In the absence of conclusive radiological diagnosis or if a patient is too unstable for imaging, bedside diagnostic laparoscopy may yield important information and negate the requirement for non-therapeutic laparotomy. Alemanno et al. recently reviewed 129 patients that underwent bedside diagnostic laparoscopy within their ICU, including 25 patients with unexplained sepsis. Findings were compared against 154 ICU patients who underwent exploratory laparotomy in the operating suite. They conclude bedside diagnostic laparoscopy in the ICU setting can be considered an option for cases with suggestive, but non-conclusive, laparotomy or radiological results, or in the rare case in which it is unsafe to transfer a critically ill patient to the radiology department [160]. Although large scale studies will be difficult to justify, diagnostic laparoscopy either at the bedside or in the operating department remains a consideration in individual cases.

## Conclusions

The prompt recognition and targeted treatment of sepsis is essential to improve clinical outcomes. We have considered the role of screening tools, clinical assessment, biomarkers, microbiology and imaging in the diagnosis of infection and highlighted the strengths and limitations associated with each. To date, there is no diagnostic tool able to rapidly identify or exclude infection. The lack of a reliable diagnostic tool contributes to the high quantities of broad-spectrum antimicrobial therapy used globally and is likely to lead to under-recognition (and over-treatment) of non-infectious inflammatory states. Given the increasing prevalence of antibiotic resistance, there is an ever-pressing need for novel solutions. Combining machine learning models with biomarkers, gene markers and EMR data may improve the utility of screening tools but requires further work. The identification and management of sepsis continues to rely on thorough patient assessment and sound clinical judgement. Novel techniques should complement, not replace, these fundamental elements of patient care.
